# Supplementary material for: Novel Susceptibility Genes Drive Familial Non-Medullary Thyroid Cancer in a Large Consanguineous Kindred
Source: Int J Mol Sci. 2023 May 4;24(9):8233. doi: 10.3390/ijms24098233 (PMC10179265; doi:10.3390/ijms24098233)
Supplement: Supplementary file 1 [file ijms-24-08233-s001.zip › ijms-2345926-supplementary.pdf]

Supplementary-

Supplementary Table S1: List of the genes reported to have mutations causing familial non-medullary thyroid cancer.

Gene	Protein	OMIM	Reference
ANXA3	Annexin A3	106490	[1]
ATM	Ataxia Telangiectasia Mutated	607585	[2]
BRCA1	Breast cancer type 1 susceptibility protein	113705	[2]
CHEK2	Checkpoint Kinase 2	604373	[2,3]
CIAO3	Cytosolic Iron-Sulfur Assembly Component 3	611118	[4]
DICER1	Endoribonuclease Dicer	606241	[2,4]
EIF1AX	Eukaryotic Translation Initiation Factor 1A X-Linked	300186	[3]
FKBP10	Peptidyl-prolyl cis-trans isomerase	607063	[1]
FOXE1	Forkhead box protein E1	602617	[2,4]
HABP2	Hyaluronan Binding Protein 2	603924	[2,4,5]
MYH9	Myosin Heavy Chain 9	160775	[2]
NKX2.1	Homeobox protein Nkx-2.1	600635	[2,4,6]
NMTC1	Non-medullary thyroid carcinoma 1	606240	[4]
NTN4	Netrin 4	610401	[1]
P2RX5	Purinergic Receptor P2X 5	602836	[1]
PLEKHG5	Pleckstrin Homology And RhoGEF Domain Containing G5	611101	[1]
PPM1D	Protein Phosphatase, Mg2+/Mn2+ Dependent 1D	605100	[3]
PTCSC2	Papillary Thyroid Carcinoma Susceptibility Candidate 2	44086*	[2]
PTCSC3	Papillary Thyroid Carcinoma Susceptibility Candidate 3	614821	[2]
RASAL1	RAS Protein Activator Like 1	604118	[2]
SAPCD1	Suppressor APC Domain Containing 1	13938*	[1]
SERPINA1	Serpin Family A Member 1	107400	[1]
SLC34A2	Solute Carrier Family 34 Member 2	604217	[7]
SPRY4	Sprouty RTK Signaling Antagonist 4	607984	[8]
SRGAP1	SLIT-ROBO Rho GTPase Activating Protein 1	606523	[2,4]
SRRM2	Serine/Arginine Repetitive Matrix 2	606032	[2,9]
TCO	Thyroid carcinoma, non-medullary, with cell oxyphilia	603386	[4]
TRPM3	Transient Receptor Potential Cation Channel Subfamily M Member 3	608961	[6]
WDR77	WD Repeat Domain 77	611734	[10]
XRCC1	X-Ray Repair Cross Complementing 1	194360	[2]
**OMIM: Online Mendelian Inheritance in Man. *HUGO Gene Nomenclature Committee (HGNC)**			

Supplementary Table S2: Family segregation analysis of the 50 negated candidate variants while comparing exome sequencing of patients – III8, III10, IVA5, XFIII4, and XFIII5.

Gene Symbol	Position	Ref/Alt	III1 A	III2 H	III4 H	III5 A	III6 H	III8 A	III10 A	IVA5 A	XFIII1 A	XFIII4 A	XFIII5 A
MSH6	chr2:48033423	A>T	-/+	+/+	+/+	-/+	-/-	-/+	-/+	-/+	-	+/+	+/+
LATS1	chr6:150023177	A>G	+/+	-	-	+/+	-	-/+	-/+	-/+	-	+/+	+/+
ITGAX	chr16:31388180	G>A	-/+	+/+	+/+	+/+	+/+	-/+	-/+	-/+	-	+/+	+/+
GNAS	chr20:57429715	A>C	-	-	-	+/+	-	+/+	+/+	+/+	-	+/+	+/+
F7	chr13:113772751	A/G	-/+	-/+	-/+	-/+	+/+	-/+	-/+	-/+	-	+/+	-/+
ATM	chr11:108172382	G/C	-/+	-	-	+/+	-	-/+	-/+	-/+	-	+/+	+/+
PEX26	chr22:18567998	G/C	-/+	-	-	+/+	-	-/+	-/+	-/+	-	+/+	+/+
QSER1	chr11:32953759	A/G	+/+	-	-	+/+	-	-/+	-/+	-/+	-	-/+	-/+
HSPG2	chr1:22157748	C/G	+/+	-	-	-/+	-	-/+	-/+	-/+	-	+/+	+/+
FCHSD1	chr5:141024648	G/A	-/+	-	-	+/+	-	-/+	-/+	-/+	-	+/+	+/+
FAM160A1	chr4:152559873	A/T	-/+	-	-	+/+	-	-/+	+/+	-/+	-	+/+	+/+
PIEZO2	chr18:10677856	C/T	-/+	-/+	-/+	-/+	-/+	-/+	-/+	-/+	-	-/+	-/+
BCR	chr22:23524386	C/G	-/+	+/+	-/-	-/+	+/+	-/+	-/+	-/+	-	+/+	+/+
ATR	chr3:142268341	C>T	-	-	-	-	-	+/+	+/+	+/+	-	+/+	+/+
RSC1A1	chr1:15986365	T>C	-	-	-	-	-	+/+	+/+	+/+	-	+/+	+/+
TERT	chr5:1294163	G>A	-	-	-	-	-	+/+	+/+	+/+	-	+/+	+/+
CUL9	chr6:43152097	C>T	-	-	-	-	-	+/+	+/+	+/+	-	+/+	+/+
SOCS5	chr2:46985961	A>C	-	-	-	-	-	+/+	+/+	+/+	-	+/+	+/+
BRCA2	chr13:32913690	C>T	-	-	-	-	-	+/+	+/+	+/+	-	+/+	+/+
CLDN10	chr13:96212695	G>A	-	-	-	-	-	+/+	+/+	+/+	-	+/+	+/+
SHPRH	chr6:146269484	delGAA	-	-	-	+/+	-	-/+	-/+	-/+	-	-/+	+/+
NID2	chr14:52505546	G>A	-	-	-		-	-/+	-/-	-/+	-	-/+	+/+
FMO2	chr1:171173065	C>T	-/+	-	-	+/+	-	-/+	-/+	-/+	-	-/+	-/+
PRKAG3	chr2:219695056	T>A	-/-	-/+	-/+	-/+	-/+	-/+	-/+	-/+	-/-	-/+	-/+
ABCA12	chr2:215919360	G>T	-/-	-/+	-/+	-/+	-/+	-/+	-/+	-/+	-/-	-/+	-/+
CCDC108	chr2:219868816	A>G	-/-	-/+	-/+	-/+	-/+	-/+	-/+	-/+	-/-	-/+	-/+
NDST4	chr4:115898360	G>C	-/+	+/+	-/+	-/+	+/+	-/+	-/+	-/+	-/+	-/+	+/+
CENPE	chr4:104030085	G>A	-/+	-	-	-	-	-/+	-/+	-/+	-/+	-/+	+/+
DCHS2	chr4:155163847	T>A	-/+	-/+	-	+/+	+/+	-/+	+/+	-/+	+/+	-/+	-/+
MDFI	chr6:41613864	C>T	-/+	-	-	-/+	-	-/+	+/+	-/+	-	-/+	+/+
PRRG4	chr11:32874843	T>A	+/+	+/+	+/+	+/+	+/+	-/+	-/+	-/+	+/+	-/+	-/+
QSER1	chr11:32953759	A>G	+/+	-	-	+/+	-	-/+	-/+	-/+	-	-/+	-/+
TBX5	chr12:114793375	G>A	-/+	-/+	-/+	-/+	-/+	-/+	-/+	-/+	+/+	-/+	-/+
SPNS2	chr17:4402454	G>T	-/+	-/+	-/+	-/+	-/+	-/+	-/+	-/+	-/+	-/+	-/+
STK11IP	chr2:220470710	G>A	-/-	-/+	-/+	-/+	-/+	-/+	-/+	-/+	-/-	-/+	-/+
SMOC2	chr6:169064783	A>G	-/+	-/-	-/+	-/-	-/+	-/-	-/+	-/-	-/+	-/+	-/+
SLC26A7	chr8:92375732	A>T	-/+	-	-	+/+	-	-/+	-/+	-/+	+/+	-/+	-/+
HECW2	chr2:197183394	G>C	-/+	-/-	-/-	-/-	-/+	-/-	-/-	-/+	-/+	-/+	-/+
TP53I13	chr17:27899895	C>T	-/+	-	-	-/-	-	-/+	-/+	-/+	+/+	-/+	-/+
LOXL2	chr8:23225582	G>A	-/+	-	-	+/+	-	-/+	+/+	-/+	-/+	-/+	-/+

GPR124	chr8:37697775	C>T	-/+	-	-	+/+	-	-/+	+/+	-/+	-/+	-/+	-/+
EFCAB5	chr17:28380799	delAG	-/+	-	-		-	-/+	-/+	-/+	+/+	-/+	-/+
CBFA2T3	chr16:88947694	G>A	-/+	-	-	+/+	-	-/+	-/+	-/+	+/+	-/+	-/+
SLC28A2	chr15:45559861	C>T	-/+	-	-	-/+	-	-/+	+/+	-/-	-/+	-/+	-/+
PPP6R2	chr22:50876272	T>C	-/+	-	-	+/+	-	-/+	-/+	-/+	-/+	-/+	+/+
CFAP65	chr2:219868816	A>G	-/-	-/+	-/+	-/+	-/+	-/+	-/+	-/+	-/-	-/+	-/+
MSRB3	chr12:65857067	G>A	-/+	-	-	-/+	-	-/+	-/+	-/-	+/+	-/+	-/+
KIF24	chr9:34269296	A>G	-/+	-	-	-/+	-	-/+	-/+	-/+	+/+	-/+	-/+
FIGNL2	chr12:52214855	A>G	-/+	-	-	-/+	-	-/+	-/+	-/-	+/+	-/+	-/+
SPAG5	chr17:26911215	C>A	-/+	-	-	-/-	-	-/+	-/+	-/+	+/+	-/+	-/+

The family members' annotations are according to Figure 1. −/−: homozygote for the mutation; −/+: heterozygote, and +/+: normal. "A" represents affected PTC individuals, and "H" represents healthy individuals. Positions on chromosomes are according to GRCh37/hg19.

Supplementary Table S3: The analysis of the protein-protein interactions (PPI) detected by the Biological General Repository for Interaction Datasets (BioGRID).

Protein	Interactor	Organism	Experimental Evidence	Reference
**ARHGEF28**	**SQSTM1**	H. sapiens	Affinity Capture-Western	[11]
**ARHGEF28**	**MYH9**	H. sapiens	Proximity Label-MS	[12]
**SQSTM1**	**TP53**	H. sapiens	Affinity Capture-Western<br>Reconstituted Complex	[13–17]
**Affinity Capture-Western: An interaction is inferred when a bait protein is affinity captured from cell extracts by either polyclonal antibody or epitope tag and the associated interaction partner identified by Western blot with a specific polyclonal antibody or second epitope tag. Proximity Label-MS: An interaction is inferred when a bait-enzyme fusion protein selectively modifies a vicinal protein with a diffusible reactive product, followed by affinity capture of the modified protein and identification by mass spectrometric methods. Reconstituted Complex: An interaction is detected between purified proteins in vitro.**				

Supplementary Table S4: The primers that were used for the PCR amplification.

Primers	Tm (c⁰)	Product size (bp)
HABP2-F (1): 5' CCCCGAGAACTCACAGTCAT 3'<br>HABP2-R (1): 5' CTGGTGATATTGTGCAAATGCT 3'	62	646
HABP2-F (2): 5' AGACTTGGGACCGCTTTCTT 3'<br>HABP2-R (2): 5' GTGCAGACAGTTGGGCAGT 3'	62	492
MSH6-F: 5' AACCGATGTTGCTTTTCTGTC 3'<br>MSH6-R: 5' CCATGCATGCCTTAAGAAAGA 3'	62	355
LATS1-F: 5' GTCCTTCGTGTGGGCTACAT 3′<br>LATS1-R: 5' GTCGAGGATCTTCGGTTGAC 3'	62	224
GNAS-F: 5' GGCAACCCCAGAAGATCC 3′<br>GNAS-R: 5' TCTGAGATGGATCTTGCGTCT 3'	63	373
ITGAX-F: 5' TCACTACTTCGTGGCGTGTC 3′<br>ITGAX-R: 5' GAAGGTGATCTGAGGAAAAGGA 3'	62	328
ATM-F: 5' CAGTGGAGGTTAACATTCATCAAG 3′<br>ATM-R: 5' GTCATGACCCACAGCAAACA 3'	63	284
F7-F: 5' CCAGATTCACCCCAGTTCAC 3′<br>F7-R: 5' CACCACATGGTCAGTGAGGA 3'	63	364
PEX26-F: 5' ACATCCCTGAAGCTGTGCTC 3′<br>PEX26-R: 5' GAGACTCCCCTCCCCAGTAG 3'	63	288
QSER1-F: 5' CAACATACCGCTCAGCTCAA 3′<br>QSER1-R: 5' GGAGTGCTGTGAACCAGTCA 3'	62	239
HSPG2-F: 5' GAGCAGCGGCTTCATAGgt 3′<br>HSPG2-R: 5' GCCGAGAACATTCAGTACCC 3'	62	364
FCHSD1-F: 5' GCCCCATATGAGGTCTGTTC 3′<br>FCHSD1-R: 5' CACCCCCATTCATACCTGAT 3'	62	177
FAM160A1-F: 5' CGTTCCAACATCTGAACTAGAATG 3′<br>FAM160A1-R: 5' TTGGCGCCTTTTTAGCTTAT 3'	61	249
FBXW10-F: 5' TGGGATCAAAACCGAGTCAT 3′<br>FBXW10-R: 5' TTCTTCCATGAGCTGCCTCT 3'	62	265
PIEZO2-F: 5' CCAATGAAAACGTGGTGACA 3′<br>PIEZO2-R: 5' CTGTGCTGTTGCCTGCTATC 3'	63	220
BCR-F: 5' CAACAGTCCTTCGACAGCAG 3′<br>BCR-R: 5' ACGTGGCGTGAGGAACTC 3'	62	177
ATR-F: 5' GCTTCTGCTCTCATTCGAACTT 3′<br>ATR-R: 5' CACCCTCTTTCCTAGAAGAATGTT 3'	62	229
CLDN10-F: 5' CACTAACCATAGTGCTTTCCCTCT 3′<br>CLDN10-R: 5' GGAAATTCATGTCTCTTGGTTGA 3'	62	199
NR4A1-F: 5' TATATGTCGGGGTCCTGGAA 3′<br>NR4A1-R: 5' TGCCTAATGCAGACATCACC 3'	62	220
CUL9-F: 5' ATTCCACCCCCTCCATGTAT 3′<br>CUL9-R: 5' CCACACTTCAGGACACTCCA 3'	62	220

SOCS5-F: 5' GCACTGCCTTAGCTACCTTCA 3′<br>SOCS5-R: 5' CAGCATCCAATGAACTCTGG 3'	62	498
TERT-F: 5' AGTGCCAGCCGAAGTCTG 3′<br>TERT-R: 5' GTACACCGGGGGACAAGG 3'	63	297
RSC1A1-F: 5' AACCCGAGTTTGAGGACCTT 3′<br>RSC1A1-R: 5' GAGCGAGCAAGACTCATAGGAT 3'	62	245
BRCA2-F: 5' GTCCTGCAACTTGTTACACAAATCA 3′<br>BRCA2-R: 5' AGCTAGTCACAAGTTCCTCAACG 3'	63	501
SHPRH-F: 5' TGTGGCTTTTCCAGAATGAGA 3′<br>SHPRH-R: 5' AATTTTGCCTTACCAGCCTGT 3'	63	237
NID2-F: 5' CTCTCTGACTTTTGGTGCAAT 3′<br>NID2-R: 5' GCATGGCACTAGGTAACCCTTA 3'	61	300
STK11IP-F: 5' GGTCTTTCTGCTGCAACCTC 3′<br>STK11IP-R: 5' CTGATGCATTCCCATCTCAC 3'	62	218
HECW2-F: 5' CGAAGGGTCGCAGGAATC 3′<br>HECW2-R: 5' CTCGAGCCAAGAGCCACAG 3'	65	293
SLC26A7-F: 5' ATCCCCGAATATCCCAGAGA 3′<br>SLC26A7-R: 5' TGCTTTTGTCTGAATGCTGAA 3'	62	475
SMOC2-F: 5' AGTGATGTGGACTCCTTCCTG 3′<br>SMOC2-R: 5' TTCTTGGATTTTCTTGGAAGGT 3'	61	242
FMO2-F: 5' CTAAACGGGACAATGCCCTA 3′<br>FMO2-R: 5' CAACTTTGCTCACCAACGAG 3'	62	300
PRKAG3-F: 5' TGTGAGATGAATGGCGACAG 3′<br>PRKAG3-R: 5' GTTTCCGCAGTTCGTCATC 3'	63	387
ABCA12-F: 5' CCCTGGAATCTGCCACATAA 3′<br>ABCA12-R: 5' TGTTCCATAAGGCTTGTTCTGA 3'	63	221
CCDC108-F: 5' TTAATGCCAATCCTGCCTGT 3′<br>CCDC108-R: 5' CCTTCTCGTCCTGTTCTTGC 3'	62	364
NDST4-F: 5' AGAGGCATCCCAAAACTTCA 3′<br>NDST4-R: 5' CAATCTGGAGAATCTATCTCTACCACT 3'	62	245
CENPE-F: 5' GCATCTTTCCAATGAGGTCAA 3′<br>CENPE-R: 5' TAGCGAACTGGATGAGGTGA 3'	63	246
DCHS2-F: 5' TTGATTGTCCAAGCCACAGA 3′<br>DCHS2-R: 5' AGTTTGCTTGGTTGTTGAGGA 3'	62	246
MDFI-F: 5' GCTCACTCCCAGATTGTGGT 3′<br>MDFI-R: 5' GACGTCGAGGTCAGTGCTGT 3'	64	365
PRRG4-F: 5' TCAAAGCTCTTGGTCTTTCCA 3′<br>PRRG4-R: 5' CCCGGCCTATCAAATAACAC 3'	62	460
TBX5-F: 5' TGGACAGGCTACCCTACCAG 3′<br>TBX5-R: 5' CATCCAGCGACCTTGAGTG 3'	63	457
SPNS2-F: 5' AGGAGGAGGAGGCGGACG 3′<br>SPNS2-R: 5' CGACAGCGTCTGCACCTC 3'	66	140
TRIOBP-F: 5' CAATAGAGCCACACGAGACAA 3′<br>TRIOBP-R: 5' GTTGTCTCGTGCAGCTCTATTG 3'	62	238

TP53I13-F: 5' ACACGGCTGCACAGAAACTT 3′<br>TP53I13-R: 5' GTCCACGTGGCAGAGGTC 3'	63	378
LOXL2-F: 5' CAACGTGGCCAAGATTCAG 3′<br>LOXL2-R: 5' GCACTCAGATCCAAAGCACA 3'	63	233
GPR124-F: 5' GGAGGGTCCAGTCGTAGTCC 3′<br>GPR124-R: 5' CAGAAGCCCGCAAGATGT 3'	63	389
EFCAB5-F: 5' AGGACAGCACAAAGGGTCAA 3′<br>EFCAB5-R: 5' GGTCCTTGTTCTGATTTTTGG 3'	62	237
CBFA2T3-F: 5' GACGCCGAGGACACAAAG 3′<br>CBFA2T3-R: 5' GCCCGTGTTTTCCTACAAAG 3'	63	239
SLC24A4-F: 5' GGATGTCATCATGGGCATTAC 3′<br>SLC24A4-R: 5' CTCCACAGCAGGACTTGACA 3'	62	208
SLC28A2-F: 5' TGGGAGCCTTCATAGCCTTT 3′<br>SLC28A2-R: 5' GCTTTACCCCCTCCTCACTC 3'	63	229
PPP6R2-F: 5' GGGAGTGCCGTCTGATGT 3′<br>PPP6R2-R: 5' ACAGGCCCATGACAGACC 3'	63	298
TMX4-F: 5' TCTGATTCCAGCATCTTCACA 3′<br>TMX4-R: 5' GAAAGCAGTCTCAAACAAGCAA 3'	62	264
TBX5-F: 5' GTGTGGGCCTCAGACTGG 3′<br>TBX5-R: 5' GCGACCTTGAGTGCAGATGT 3'	64	297
CFAP65-F: 5' TTAATGCCAATCCTGCCTGT 3′<br>CFAP65-R: 5' CCTTCTCGTCCTGTTCTTGC 3'	62	364
MSRB3-F: 5' CATGCTTTTCCCTGACGTTT 3′<br>MSRB3-R: 5' CACTTTGTTTCCATCACTCTCC 3'	62	232
ARHGEF28-F: 5' CCTCTGCTCGGAAGGTTACTC 3′<br>ARHGEF28-R: 5' AGAACTTCCCAACACAGTCCA 3'	62	247
KIF24-F: 5' CACTCCTGCTGTGTCTTTTCTC 3′<br>KIF24-R: 5' CAGTTGCTTTGCTGATTCCA 3'	62	233
FIGNL2-F: 5' GACGAGCAAGATGGTGGACT 3′<br>FIGNL2-R: 5' CTCGCTGATGAGGAGTACGG 3'	63	343
SLC47A1-F: 5' CATGGGGAGGTAATGACTGC 3′<br>SLC47A1-R: 5' CAGCAGATCATGTGCAGTTTC 3'	62	600
SPAG5-F: 5' GCATCCAGGATTTAGGACCA 3′<br>SPAG5-R: 5' CCCTGGGTACAGAGGCTTTT 3'	62	268
**Tm, melting temperature. F, forward primer. R, reverse primer.**		

SUPPLEMENTARY REFERENCES

1. Sarquis, M.; Moraes, D.C.; Bastos-Rodrigues, L.; Azevedo, P.G.; Ramos, A.V.; Reis, F.V.; Dande, P.V.; Paim, I.; Friedman, E.; De Marco, L. Germline Mutations in Familial Papillary Thyroid Cancer. *Endocr. Pathol.* **2020**, *31*, 14–20. https://doi.org/10.1007/s12022-020-09607-4.
2. Hińcza, K.; Kowalik, A.; Kowalska, A. Current Knowledge of Germline Genetic Risk Factors for the Development of Non-Medullary Thyroid Cancer. *Genes* **2019**, *10*, 482. https://doi.org/10.3390/genes10070482.
3. Kandoth, C.; Schultz, N.; Cherniack, A.D.; Akbani, R.; Liu, Y.; Shen, H.; Levine, D.A. Integrated Genomic Characterization of Papillary Thyroid Carcinoma. *Cell* **2014**, *159*, 676–690. https://doi.org/10.1016/j.cell.2014.09.050.
4. Yang, S.P.; Ngeow, J. Familial non-medullary thyroid cancer: Unraveling the genetic maze. *Endocr. Relat. Cancer* **2016**, *23*, R577–R595. https://doi.org/10.1530/erc-16-0067.
5. Gara, S.K.; Jia, L.; Merino, M.J.; Agarwal, S.K.; Zhang, L.; Cam, M.; Patel, D.; Kebebew, E. Germline *HABP2* Mutation Causing Familial Nonmedullary Thyroid Cancer. *New Engl. J. Med.* **2015**, *373*, 448–455. https://doi.org/10.1056/nejmoa1502449.
6. Hwangbo, Y.; Lee, E.K.; Son, H.-Y.; Im, S.-W.; Kwak, S.-J.; Yoon, J.W.; Kim, M.J.; Kim, J.; Choi, H.S.; Ryu, C.H.; et al. Genome-Wide Association Study Reveals Distinct Genetic Susceptibility of Thyroid Nodules From Thyroid Cancer. *J. Clin. Endocrinol. Metab.* **2018**, *103*, 4384–4394. https://doi.org/10.1210/jc.2017-02439.
7. He, J.; Zhou, M.; Li, X.; Gu, S.; Cao, Y.; Xing, T.; Chen, W.; Chu, C.; Gu, F.; Zhou, J.; et al. SLC34A2 simultaneously promotes papillary thyroid carcinoma growth and invasion through distinct mechanisms. *Oncogene* **2020**, *39*, 2658–2675. https://doi.org/10.1038/s41388-020-1181-z.
8. Marques, I.J.; Gomes, M.I.; Pojo, M.; Pires, M.C.; Moura, M.M.; Cabrera, R.; Santos, C.; van Ijcken, W.F.J.; Teixeira, M.R.; Ramalho, J.S.; et al. Identification of *SPRY4* as a Novel Candidate Susceptibility Gene for Familial Nonmedullary Thyroid Cancer. *Thyroid* **2021**, *31*, 1366–1375. https://doi.org/10.1089/thy.2020.0290.
9. Tomsic, J.; He, H.; Akagi, K.; Liyanarachchi, S.; Pan, Q.; Bertani, B.; Nagy, R.; Symer, D.E.; Blencowe, B.J.; de la Chapelle, A. A germline mutation in SRRM2, a splicing factor gene, is implicated in papillary thyroid carcinoma predisposition. *Sci. Rep.* **2015**, *5*, 10566. https://doi.org/10.1038/srep10566.
10. Zhao, Y.; Yu, T.; Sun, J.; Wang, F.; Cheng, C.; He, S.; Chen, L.; Xie, D.; Fu, L.; Guan, X.; et al. Germ-line mutations in *WDR77* predispose to familial papillary thyroid cancer. *Proc. Natl. Acad. Sci. USA* **2021**, *118*, e2026327118. https://doi.org/10.1073/pnas.2026327118.
11. Keller, B.A.; Volkening, K.; Droppelmann, C.A.; Ang, L.C.; Rademakers, R.; Strong, M.J. Co-aggregation of RNA binding proteins in ALS spinal motor neurons: Evidence of a common pathogenic mechanism. *Acta Neuropathol.* **2012**, *124*, 733–747. https://doi.org/10.1007/s00401-012-1035-z.
12. Fasci, D.; van Ingen, H.; Scheltema, R.A.; Heck, A.J.R. Histone Interaction Landscapes Visualized by Crosslinking Mass Spectrometry in Intact Cell Nuclei. *Mol. Cell Proteom.* **2018**, *17*, 2018–2033. https://doi.org/10.1074/mcp.ra118.000924.
13. Horikawa, I.; Fujita, K.; Jenkins, L.M.M.; Hiyoshi, Y.; Mondal, A.M.; Vojtesek, B.; Lane, D.P.; Appella, E.; Harris, C.C. Autophagic degradation of the inhibitory p53 isoform Δ133p53α as a regulatory mechanism for p53-mediated senescence. *Nat. Commun.* **2014**, *5*, 4706. https://doi.org/10.1038/ncomms5706.
14. Kang, J.H.; Lee, J.-S.; Hong, D.; Lee, S.-H.; Kim, N.; Lee, W.-K.; Sung, T.-W.; Gong, Y.-D.; Kim, S.-Y. Renal cell carcinoma escapes death by p53 depletion through transglutaminase 2-chaperoned autophagy. *Cell Death Dis.* **2016**, *7*, e2163. https://doi.org/10.1038/cddis.2016.14.
15. Mukherjee, S.; Maddalena, M.; Lü, Y; Martinez, S.; Nataraj, N.B.; Noronha, A.; Oren, M. Cross-talk between mutant p53 and p62/SQSTM1 augments cancer cell migration by promoting the degradation of cell adhesion proteins. *Proc. Natl. Acad. Sci. USA* **2022**, *119*, e2119644119.
16. Yuan, F.; Sun, Q.; Zhang, S.; Ye, L.; Xu, Y.; Deng, G.; Xu, Z.; Zhang, S.; Liu, B.; Chen, Q. The dual role of p62 in ferroptosis of glioblastoma according to p53 status. *Cell Biosci.* **2022**, *12*, 20. https://doi.org/10.1186/s13578-022-00764-z.
17. Di Lello, P.; Jenkins LM, M.; Jones, T.N.; Nguyen, B.D.; Hara, T.; Yamaguchi, H.; Omichinski, J.G. Structure of the Tfb1/p53 Complex: Insights into the Interaction between the p62/Tfb1 Subunit of TFIIH and the Activation Domain of p53. *Mol. Cell* **2006**, *22*, 731–740.
